# Role of Neuro-Immune Cross-Talk in the Anti-obesity Effect of Electro-Acupuncture

**DOI:** 10.3389/fnins.2020.00151

**Published:** 2020-02-28

**Authors:** Mengjiang Lu, Yan He, Meirong Gong, Qian Li, Qianqian Tang, Xuan Wang, Yaling Wang, Mengqian Yuan, Zhi Yu, Bin Xu

**Affiliations:** ^1^Key Laboratory of Acupuncture and Medicine Research of Ministry of Education, Nanjing University of Chinese Medicine, Nanjing, China; ^2^The Affiliated Hospital of Nanjing University of Chinese Medicine, Nanjing, China

**Keywords:** obesity, electroacupuncture, inguinal white adipose tissue, sympathetic nervous system, sympathetic associated macrophage

## Abstract

There is evidence to show that electro-acupuncture (EA) has a promotive effect on both lipolysis and thermogenesis, and that these mechanisms underlie the anti-obesity effect of EA. The sympathetic nervous system (SNS) is known to play a role in thermogenesis. Additionally, obesity is characterized by a chronic low-grade inflammatory state. Based on these findings, the aim of the present study is to investigate the potential neuro-immune mechanisms underlying the therapeutic effect of EA in obesity. In the experiment, we used a high fat diet (HFD) rats model to study the effect of EA in reducing body weight. EA increases the activity of sympathetic nerves in inguinal white adipose tissue (iWAT), especially in the HFD group. Compared to HFD rats, EA can decrease sympathetic associated macrophage (SAM) and the level of norepinephrine transporter protein (Slc6a2). The relative uncoupling protein 1 expression shows EA increases thermogenesis in iWAT, and increases β3 receptors. Interestingly, injecting β antagonist in iWAT increases Slc6a2 protein levels. Additionally, the SNS-macrophage cross-talk response to EA showed in iWAT but not in epididymis white adipose tissue. The results of the present study indicate that EA exerts its anti-obesity effect via three mechanisms: (1) inhibition of SAMs and the norepinephrine transporter protein SlC6a2, (2) promoting SNS activity and thermogenesis, and (3) regulating immunologic balance.

## Introduction

Obesity is a growing global problem. In 2017, obesity was defined as a chronic, relapsing disease by the World Obesity Federation (Bray et al., 2017). Multiple pharmacotherapies are approved by the FDA for the treatment of obesity. However, their efficiency and safety are limited and, therefore, several weight loss drugs are now off the market (Christensen et al., 2007; James et al., 2010). Bariatric surgery is appropriate for all patients with BMI (kg/m^2^) > 40 and for patients with associated comorbid conditions (Wolfe et al., 2016). The high surgical risk is a limitation of bariatric surgery (Flum and Dellinger, 2004; Smith et al., 2011). There are also many complementary and alternative approaches to obesity treatment, and acupuncture is one of the most widely used among them. There is reported evidence that acupuncture is effective and safe for the treatment of simple obesity (Zhang et al., 2017). In particular, there is experimental evidence that acupuncture not only reduces body weight, but also increases adiponectin and decreases leptin in obese rats (Peplow, 2015). This is supported by the results of clinical trials which show that acupuncture can reduce not only body weight and fat content, but also obesity-related complications, such as serum pro-oxidant antioxidant imbalance, dyslipidemia, and inflammation (Mazidi et al., 2017). Another interesting experimental finding is that electro-acupuncture (EA) increases cold endurance in the Stat5NKO mouse model of obesity (Fu et al., 2017). This is supported by other evidence which shows that acupuncture not only controls appetite but also increases thermogenesis, which is a mechanism for heat generation and, therefore, cold endurance (Du et al., 2013; Martin, 2019). Further research shows thermogenesis is related to PGC-1 α/UCP-1 signaling in WAT (Wang et al., 2018). As adipose tissue plays an important role in thermogenesis, it is possible that the anti-obesity effect of EA is associated with UCP-1 signal-related mechanisms.

On exposure to cold, white adipose tissue is transformed into brown adipose tissue in a process that is referred to as fat browning, which involves the uncoupling of oxygen consumption from ATP synthesis in the mitochondria (Loncar et al., 1988; Klingenspor, 2003). Research has shown that the sympathetic nervous system (SNS) plays an important role in fat browning (Kooijman et al., 2015). Stimulation of the SNS in adipose is associated with the release of noradrenaline (NE), and NE along with the β-adrenergic receptor leads to mitochondrial uncoupling of protein 1 and (UCP1)-dependent uncoupling of oxygen consumption (Francois et al., 2018). Other studies have shown that alternatively activated macrophages secrete catecholamines to induce the expression of thermogenic genes in brown adipose tissue and lipolysis-related genes in white adipose tissue (Nguyen et al., 2011). Additionally, one study showed that sympathetic adipose macrophages (SAMs) are involved in the reduction of thermogenesis through their role in the transport of NE in inguinal white adipose tissue (iWAT) via solute carrier family 6 member 2 (Slc6a2) (Pirzgalska et al., 2017). Thus, thermogenesis in the adipose may be dependent on the interaction between neurons and immunocytes. Based on these findings, we assumed EA reduced body weight via the browning of WAT, and that the therapeutic effect of EA in obesity involves these neuro-immune cross-talk mechanisms.

In the present study, we have confirmed the therapeutic effect of EA in rats with high-fat diet (HFD)-induced obesity, and have explored the potential neuro-immune mechanisms underlying this effect by examining the expression of the SNS, iWAT, SAMs, NE, β3 adrenergic receptor, UCP1, and Slc6a2 in obese rats that are treated with EA.

## Materials and Methods

### Animals

The experiment animals were 65 weaning (3 weeks) male Sprague-Dawley rats (Model Animal Research Center of Nanjing Medical University, China) that were divided into a normal diet group (the control group), an HFD group, and an HFD with EA group. The rats were housed under controlled environmental conditions (temperature: 22°C, relative humidity: 40–60%, and 12/12 h light/dark cycle) and were given free access to water and food. The HFD + EA group and HFD group were fed a diet containing 45 kcal% fat (Research Diets, D12451) providing 473 kcal/100 g, and the normal group were fed a regular diet of rat chow providing 352 kacl/100g. After feeding for 11 weeks, EA intervention was performed on the HFD + EA group. The duration of the EA intervention was 4 weeks. The animals’ body weight was measured every week. Before the sacrifice, three rats from the HFD group were injected with β receptor antagonist Propranolol in iWAT. All experiments were performed according to the Principles of Laboratory Animal Care and the Guide for the Care and Use of Laboratory Animals published by the National Science Council, China.

### EA Stimulation

ST25 (Tianshu) is located 5 mm laterally to the intersection between the upper 2/3 and the lower 1/3 (Yu et al., 2013), in the line joining the xiphoid process and the upper border of the pubic symphysis. The region of ST25 is widely used in clinical trials (Chen et al., 2018). The needles were connected to Han’s EA instrument (LH402A; Beijing Huawei Technologies Co. Ltd). The stimulation current was 2 mA; alternating frequency, 2/15 Hz; stimulation time, 20 min. The procedure was performed 6 times a week and lasted for 4 weeks. In the electrophysiology experiment, the EA stimulation lasted for 60 s.

### Electrophysiology Measurements

The rats from the normal diet group and HFD group were anesthetized with isoflurane (RWD Life Science, China), and an inguinal incision was made. Then, the nerves present in iWAT were separated and connected to double claw platinum electrodes. Firing rate was recorded using a preamplifier (NL100; CED, United Kingdom) and a Micro1401-3 bioelectric module (NL125NL126; CED, United Kingdom) connected to a biosignal acquisition and analysis system (Microl 1401-3; CED, United Kingdom). Signal filtering was set to 10–1000 Hz; the sampling frequency was 20000 Hz and the amplification was 1000 times. The data were recorded over 180 s each time with the Spike2 software: before EA, 60 s; during EA, 60 s; after EA, 60 s.

### ELISA

Standard or sample solution (100 μL) was added to the 96 well polystyrene microplates and incubated at 37°C for 90 min. Then, 10 μL of biotinylated antibodies (Nanjing Jiancheng Bioengineering Institute, Nanjing) was added to the wells and incubated at 37°C for 60 min. The solution in each well was then aspirated, and each well was washed three times with 350 μL of 1 × wash buffer. Next, 100 μL of Tetramethylbenzidine (TMB) substrate was added to each well and incubated for 10 min. To stop the reaction, 100 μL of stop solution was added, and the wells were read at 450 nm.

### Western Blot Analysis

Inguinal white adipose tissue was harvested using the Adipose Protein Extraction Kit (MinuteTM) for protein extraction. 80 μg of adipose tissue was homogenized with 300 μl extraction buffer. After centrifuging at 2,000 rpm for 1 min, the tissue was transferred to a filter cartridge with a collection tube and incubated at −20°C for 15–20 min. After incubation, it was immediately centrifuged at 2000 rpm for 1–2 min with cap open. The flow-through contained total proteins from adipose tissue. Then, 20 μg of total tissue lysate was separated by SDS-PAGE (10% of separation gel and 5% of concentration gel). The electrophoresis contained 80 V for 0.5 h and 120 V for 1 h. The protein bands were transferred to a PVDF membrane using Trans-Blot (Bio-Rad), and 5% BSA was added for 2 h for blocking the membrane. The membrane was then reacted with primary antibodies against: UCP1 (1:1000, Abcam), norepinephrine transporter (1:1000, Abcam), β-actin (1:1000, Abcam), β3 adrenergic receptor (1:1000, Abcam), and tyrosine hydroxylase (1:1000, Abcam) under 4°C overnight. Incubation with the corresponding secondary antibodies (diluted to 1:3000, Abcam) was performed at room temperature for 1 h.

### Whole-Mount Immunostaining

The procedure was based on the protocol of Xin (Li et al., 2019). iWAT samples were immersed in a fixation solution (1% PFA in 1 × PBS, pH 7.4) at 4°C for 24–48 h. The samples were cut into 2-mm^3^ cubes and fixed with 1 × PBS-TX. They were then blocked with Sea Block blocking buffer (Thermo Fisher Scientific, United States) for 2 h, and incubated with primary antibodies against the following proteins at 4°C overnight: TH (1:200, Abcam), F4/80 (1:200, Santa Cruz), and the NE transporter Slc6a2 (1:200, Abcam). They were then incubated with the corresponding secondary antibodies (Alexa Fluor 488, Alexa Fluor 594, and Alexa Fluor 647, respectively; Abcam) at a dilution of 1:200 at RT for 2 h. For optical clearance, 90% glycerol was used, and the samples were maintained at 4°C in the dark until they became transparent. The images were captured by a laser scanning confocal microscope.

### RT-PCR

Total RNA was extracted from 200 mg of iWAT using Trizol (TaKaRa, Japan). The total RNA was reverse-transcribed to produce cDNA by a thermal cycler (Bio-Rad, United States) with PrimeScript RT Master Mix (TaKaRa, Japan) at 37°C for 15 min and 98°C for 15 s. The PCR reaction mixture (20 μL) contained of 4 μL of forward primer, 4 μL of reverse primer, 2 μL of cDNA, and 10 μL of SYBR Green Mix (TaKaRa, Japan). The PCR protocol was as follows: 40 cycles of amplification for 30 s at 95°C, 5 s at 95°C, and 30 s at 60°C. Data were processed using the 2^–ΔΔCT^ method. The primers used in the experiment are shown in Table 1.

**TABLE 1:** Sequences of the primers used for real-time PCR.

**Gene names**	**Primer sequence**	
GAPDH	Forward	5′-GGT GCT GAG TAT GTC GTG GAG-3′
	Reverse	5′-GTC TTC TGA GTG GCA GTG ATG-3′
IL-1b	Forward	5′-CCT CGT GCT GTC TGA CCC AT-3′
	Reverse	5′-CAA ACC GCT TTT CCA TCT TCT TC-3′
TNFα	Forward	5′-TGC CTC AGC CTC TTC TCA TTC C-3′
	Reverse	5′-TCC TCC GCT TGG TGG TTT G-3′
Il-10	Forward	5′-CCA GTC AGC CAG ACC CAC AT-3′
	Reverse	5′-AAT CAT TCT TCA CCT GCT CCA C-3′
Arg1	Forward	5′-TGG ACCCAG TAT TCA CCC C-3′
	Reverse	5′-GAT TACCTT CCC GTT TCG TT-3′

### Statistical Analysis

Data were analyzed using Prism 6.0. All the data are presented as mean ± SE. Groups were compared using one-way analysis of variance (ANOVA) followed by *post hoc* Student Newman-Keuls tests. *P* < 0.05 was considered to indicate statistical significance. The images of whole-mount immunostaining were analyzed by imaging pro plus 6.0, using Pearson’s correlation to represent colocalization.

## Results

### Effect of EA in HFD Rats

**FIGURE 1:**
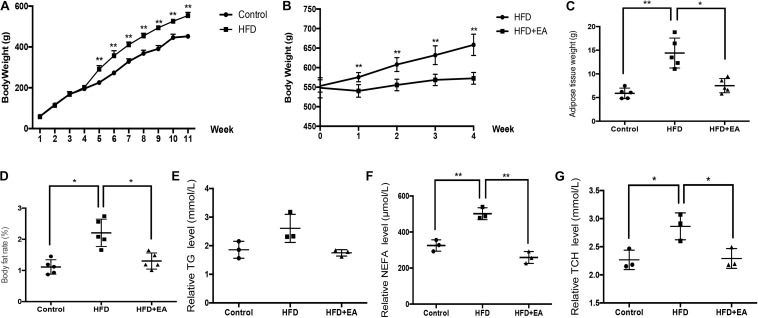
Effect of EA on body weight, adipose weight, body fat rate, and lipid levels. **(A)** Body weight of rats from the normal diet (control) and high-fat diet (HFD) groups after 11 weeks (*n* = 5, **P < 0.01). **(B)** Body weight of HFD rats with or without EA after feeding for 11 weeks and initiation of EA treatment. **(C)** White adipose tissue weight in the control group, HFD group, and HFD + EA group (*n* = 5, *P < 0.05, **P < 0.01). **(D)** Body fat rate (adipose tissue weight/body weight) of the control group, HFD group, and HFD + EA group (*n* = 5, *P < 0.05). **(E)** Relative serum TG level of the control group, HFD group, and HFD + EA group (*n* = 3). **(F)** Relative serum NEFA level of the control group, HFD group, and HFD + EA group (*n* = 3, **P < 0.01). **(G)** Relative serum TCH level of the control group, HFD group, and HFD + EA group (*n* = 3, *P < 0.05).

After feeding for 11 weeks, body weight, adipose weight, body fat rate (adipose weight divided by the total body weight), and lipid levels were significantly higher in the HFD group than in the control group (Figures 1A–F). After initiation of EA treatment, body weight was significantly lower in the HFD + EA group than in the HFD group (Figure 1B). Additionally, after 4 weeks of EA, adipose weight, body fat rate, non-esterified fatty acid (NEFA) levels, and total cholesterol (TCH) levels were significantly decreased in comparison with the HFD group without EA (Figures 1C,D,F,G). Although the HFD + EA rats had lower TG levels, the difference compared to the HFD rats was not significant (Figure 1E).

### Effect of EA on SNS Activity in iWAT

**FIGURE 2:**
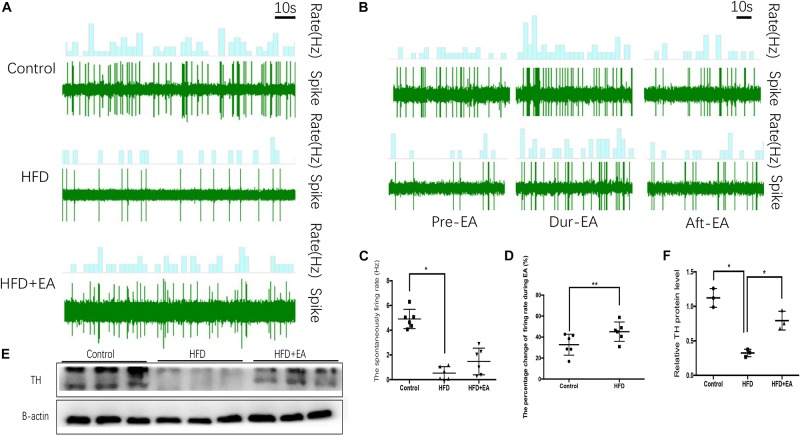
Effect of EA on SNS firing in iWAT. **(A)** Waveform representing spontaneous discharge of the SNS in iWAT (control, HFD, and HFD + EA groups). **(B)** Waveform representing spontaneous discharge during EA in the control and HFD group. **(C)** Frequency of spontaneous discharge in the control, HFD, and HFD + EA groups (*n* = 6, *P < 0.05). **(D)** Percentage change in the firing rate during EA in the control and HFD group (*n* = 6, **P < 0.01). **(E)** Immunoblot image of TH staining. **(F)** Relative protein level of TH in iWAT (in the control, HFD, and HFD + EA groups) (*n* = 3, *P < 0.05).

To evaluate SNS activity, we measured the firing rate of sympathetic nerves in iWAT. The spontaneous SNS firing rate was significantly lower in the HFD rats than in the control rats, but EA did not bring about a significant improvement in this firing rate (Figures 2A–C). During EA, the firing rate is increased in both normal and HFD rats, and the increase is particularly apparent in HFD rats (Figures 2B,D). Finally, western blot analysis of the sympathetic nerve marker TH in iWAT showed that while TH is significantly lower in HFD rats than in control rats, EA significantly increases TH expression in HFD rats (Figures 2E,F).

### Effect of EA on Inflammatory Status

**FIGURE 3:**
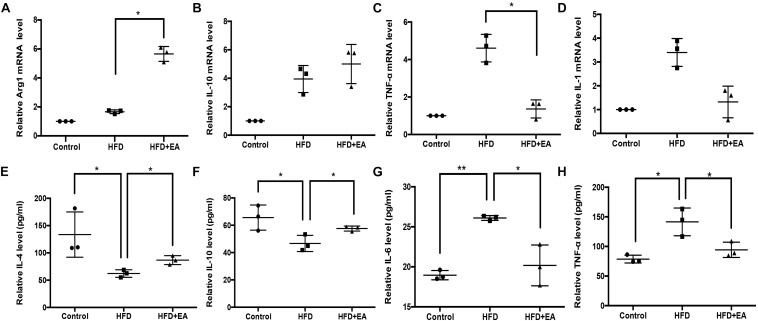
Effect of EA on the expression of inflammation levels in iWAT and blood. **(A)** Relative Arg1 mRNA level in iWAT (*n* = 3, *P < 0.05). **(B)** Relative IL-10 mRNA level in iWAT (*n* = 3). **(C)** Relative TNF-α mRNA level in iWAT (*n* = 3, *P < 0.05). **(D)** Relative IL-1 mRNA level in iWAT (*n* = 3). **(E)** Relative IL-4 protein expression in serum (*n* = 3, *P < 0.05). **(F)** Relative IL-10 protein expression in serum (*n* = 3, *P < 0.05). **(G)** Relative IL-6 protein expression in serum (*n* = 3, *P < 0.05, **P < 0.01). **(H)** Relative TNF-α protein expression in serum (*n* = 3, *P < 0.05).

High fat diet rats had lower levels of anti-inflammatory cytokines and higher levels of pro-inflammatory cytokines, in both blood and iWAT, than the control rats: the levels of IL-4, IL-10, IL-6, and TNF-α were significantly different between the two groups (Figures 3E–H). EA alleviated these effects of HFD, with significant effects observed for Arg1, IL-4, IL-10, IL-6, and TNF-α (Figures 3A–H).

### SNS–Macrophage Cross-Talk in Response to EA

**FIGURE 4:**
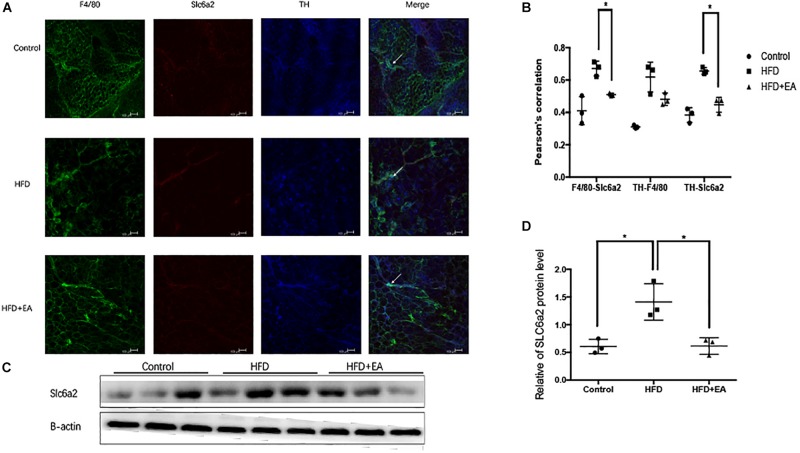
Effect of EA on SAMs. **(A)** Representative images of showing F4/80 (green), Slc6a2 (red), and TH (blue) fluorescence. The white arrows in the merged image indicate SAMs. **(B)** Correlation between F4/80 and Slc6a2, Slc6a2 and TH, and TH and F4/80, as indicated by Pearson correlation analysis (*n* = 3, *P < 0.05). **(C)** Immunoblot image of Slc6a2 staining. **(D)** Relative protein level of Slc6a2 (in the control, HFD, and HFD + EA groups) (*n* = 3, *P < 0.05).

In order to evaluate the cross-talk between the SNS and macrophages, we used whole-mount immunostaining to detect the expression of Slc6a2 (an NE transporter that is a marker of SAM), TH (an SNS marker), and F4/80 (a fluorescence marker). Colocalization of F4/80, TH, and Slc6a2 on fluorescence images was considered to be representative of SAMs in iWAT. The HFD rats showed the highest fluorescence intensity for the colocalization, and EA was found to decrease this intensity (Figure 4A). Pearson’s correlation analysis showed that the SAMs in the HFD group are higher than in the normal group and lower than in the EA group (Figure 4B). Finally, the western blot shows higher expression of Slc6a2 in the HFD group than in the EA group, which confirms that EA caused a decrease in Slc6a2 (Figures 4C,D).

### Effect of EA on Thermogenesis in iWAT and the Role of Slc6a2

**FIGURE 5:**
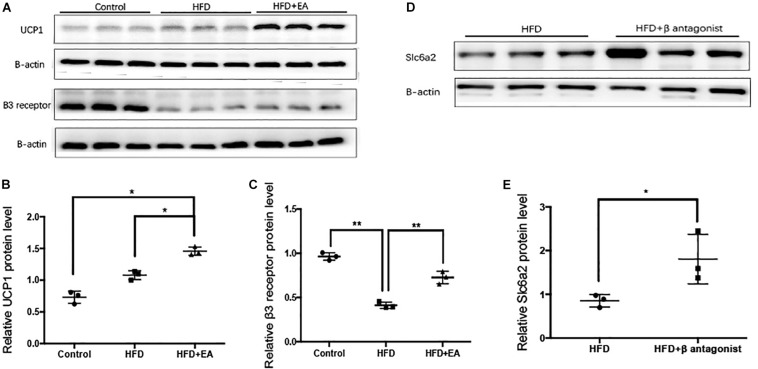
Effect of EA on the thermogenesis-related proteins UCP1 and β3 adrenergic receptor and the NE transporter Slc6a2. **(A)** Immunoblot image showing UCP1 and β3 adrenergic receptor. **(B)** Relative protein level of UCP1 (in the control, HFD, and HFD + EA groups) (*n* = 3, *P < 0.05). **(C)** Relative protein level of β3 adrenergic receptor (in the control, HFD, and HFD + EA groups) (*n* = 3, **P < 0.01). **(D)** Immunoblot image showing Slc6a2 expression in the HFD group and in the HFD group that was administered a β3 adrenergic receptor antagonist. **(E)** Relative protein level of Slc6a2 (in the HFD, and HFD + β antagonist) (*n* = 3, *P < 0.05).

The β3 adrenergic receptor and UCP1 are known to be involved in SNS-mediated thermogenesis in iWAT. Therefore, we detected the protein expression of UCP1 and the β3 adrenergic receptor in iWAT. Western blot analysis showed that EA-treated rats had significantly higher expression of both proteins than the HFD rats that did not receive EA (Figures 5A–C). We also found that HFD caused a significant decrease in β3 adrenergic receptor expression, so a β3 adrenergic receptor antagonist was injected into iWAT; it was found to cause a significant increase in Slc6a2 protein expression (Figures 5D,E).

### SNS–Macrophage Do Not Cross-Talk in Response to EA in eWAT

**FIGURE 6:**
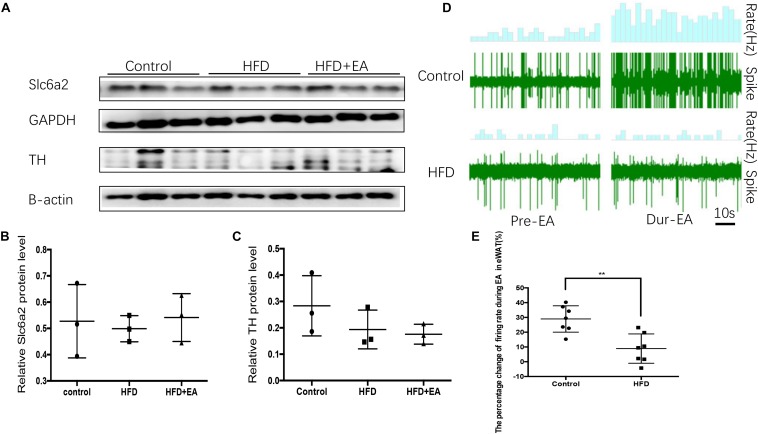
Effect of EA on the SNS-macrophage cross-talk shows in iWAT but not in eWAT. **(A)** Immunoblot image showing Slc6a2 and TH in eWAT. **(B)** Relative protein level of Slc6a2 (in the control, HFD, and HFD + EA groups) (*n* = 3, *P* > 0.05). **(C)** Relative protein level of TH (in the control, HFD, and HFD + EA groups) (*n* = 3, *P* > 0.05). **(D)** Waveform representing spontaneous discharge in eWAT during EA in the control and HFD group. **(E)** Percentage change in the firing rate of eWAT during EA in the control and HFD group (*n* = 6, **P < 0.01).

To clarify whether the SNS–macrophage cross-talk in response to EA in other white adipose tissue, we measured the expression of Slc6a2 protein in eWAT. Western blot analysis showed that the three rat groups had no significant expression of both Slc6a2 and TH protein (Figures 6A–C). We also found that during EA, the firing rate increased in the normal group but not in the HFD group (Figures 6D,E and Supplementary Data Sheet S1).

## Discussion

Our research has confirmed the therapeutic effects of EA on body weight increase and blood lipid levels in HFD-induced obese rats. Importantly, in the present research, we have clarified the neuro-immune mechanisms of EA that underlie its effect on lipolysis and thermogenesis in obese rats (Du et al., 2013; Martin, 2019).

Sympathetic efferent nerves release NE, which combines with β3 adrenergic receptors in adipocytes to drive lipolysis and thermogenesis (Mattsson et al., 2011; Bartness et al., 2014). In our research, we found a significant increase in the firing rate of the SNS in HFD rats that were treated with EA. Further, the SNS marker TH was also increased in HFD rats in response to EA treatment. These effects are indicative of an activation of SNS in iWAT induced by EA, as it has been reported that intra-adipose sympathetic firing is increased on cold-induced fat browning (which is associated with increased thermogenesis).

NE present in the adipose is known to participate in thermogenesis and lipolysis: sympathetic nerves in the adipose release NE, which combines with adrenergic receptors to activate the cAMP signal pathway, which in turn activates protein kinase-A and leads to adipose lipolysis and thermogenesis in brown and white adipose (Cannon and Nedergaard, 2004; Bowers et al., 2005; Oelkrug et al., 2015). β adrenergic receptors (β1, β2, and β3) are essential mediators for NE function in the adipose, and a lack of these three receptor subtypes is associated with metabolic dysfunction and obesity (Bachman et al., 2002). In particular, β3 adrenergic receptors have been found to be important for the control of diabetes and obesity (Komai et al., 2016). The activation of β3-adrenergic receptors leads to excitation of UCP1, uncoupling of oxidative phosphorylation and an increase in energy expenditure. In keeping with this, β3 adrenoceptor agonists have been found to significantly increase metabolic rate and lipolysis (Fisher et al., 1998). Two β3-AR agonists, BRL-37344 and CL316243, were reported to induce lipolysis and thermogenesis in brown adipocytes from rats (Atgie et al., 1997). Also, a β3-AR agonist called CGP-12177A enhanced uncoupling content in BAT and iWAT in mice. However, the lipolytic and thermogenic effects of β3-AR are controversial. The beta(1)/beta(2)/beta(3)-adrenoceptor triple knockout (TKO) mice shows beta-adrenergic signaling is essential for the resistance to obesity and cold, but not for the lipolytic response to fasting (Jimenez et al., 2002). Recent research shows intact BAT thermogenesis despite the lack of β3AR in β3ARKO mice. β3-AR is essential for lipolysis but not to brown adipose thermogenesis (Preite et al., 2016). In the present study, EA was found to increase the expression of the UCP1 and β3 adrenergic receptor in HFD rats. Thus, the anti-obesity effect of EA may involve NE-related sympathetic mechanisms.

Obesity is characterized by chronic low-grade inflammation, which is indicative of the close connection between obesity and immune mechanisms (Brestoff and Artis, 2015). It has been shown that proinflammatory macrophages accumulate in the adipose of obese mice, and that these cells are dominant sources of TNF-α, which promotes insulin resistance (Hotamisligil et al., 1993; Weisberg et al., 2003). In our study, EA was found to decrease the levels of pro-inflammatory cytokines and increase the levels of anti-inflammatory cytokines in blood and in adipose. This implies that the anti-obesity effect of EA also involves immunoregulatory mechanisms.

In classical theory, macrophages are one of the most characteristic immune cells in adipose tissue. In rodents and humans from lean to obese states, macrophage accumulation increased from 10 to 40%, respectively (Murray et al., 2014). Macrophages often exhibit a pro-inflammatory or anti-inflammatory phenotype and are conventionally classified as Ml (classic activating) and M2 (alternatingly activated) (Boutens and Stienstra, 2016). M1 macrophages secrete high levels of pro-inflammatory cytokines (such as tumor necrosis factor (TNF-α), IL-6, IL-1β) and produce reactive oxygen species through activation of inducible nitric oxide synthase (iNOS). In contrast, M2 macrophages secrete anti-inflammatory cytokines (such as IL-10, TGF-β, and IL-4). In obese states, macrophages polarized from the M2 to the M1 type (Komohara et al., 2016). The latest research showed there are not only classic pro-inflammatory and anti-inflammatory macrophages, but also other functional subtypes of macrophages (Jaitin et al., 2019). Recently, a new type of macrophage subtype called sympathetic adipose macrophages or SAMs were discovered. This macrophage is closely associated with sympathetic nerves in terms of structure and function, and it hinders the crosstalk between the adipose and sympathetic nerves. SAMs transport NE via an increase in the expression of the NE transporter Slc6a2, and this makes NE unavailable for thermogenesis-associated sympathetic signaling in the adipose. The genetic deletion of *Slc6a2* has been shown to significantly inhibit NE uptake by SAMs, and thereby enhance sympathetic signaling to the adipose tissue and improve energy homeostasis (Pirzgalska et al., 2017). Our present research showed that EA decreased the amount of SAMs in HFD rats. Further, treatment of iWAT with a β3 receptor antagonist resulted in an increase in the expression of Slc6a2. Thus, altogether, our findings indicate that the anti-obesity effect of EA is dependent on neuro-immune cross-talk mechanisms.

Cold exposure – a dominant regulator of beige adipogenesis and function – is a potential new therapy for obesity. Beige adipogenesis in WAT may reduce weight by thermogenesis (Xue et al., 2005). However, there are many differences between subcutaneous white adipose tissue (sWAT) and other white adipose tissue like epididymal adipose tissue (eWAT). Upon cold exposure, the expression of thermogenesis-relative protein increased in sWAT but not in eWAT (Jia et al., 2016). Our research showed that in obese states, SNS activity increased in iWAT but not in eWAT. Recruitment of brown/beige adipocytes (BAs) in white adipose tissue (WAT) closes interactions with resident immune cells. The single-cell RNA sequencing (scRNA-seq) identified different macrophage sub-populations in iWAT and eWAT (Burl et al., 2018). Moreover, a recent research showed in diet-induced obesity, GABA reduced monocyte migration in iWAT, but not in eWAT (Hwang et al., 2019). Our research showed SNS-macrophage cross-talk in response to EA via iWAT but not eWAT.

## Conclusion

**FIGURE 7:**
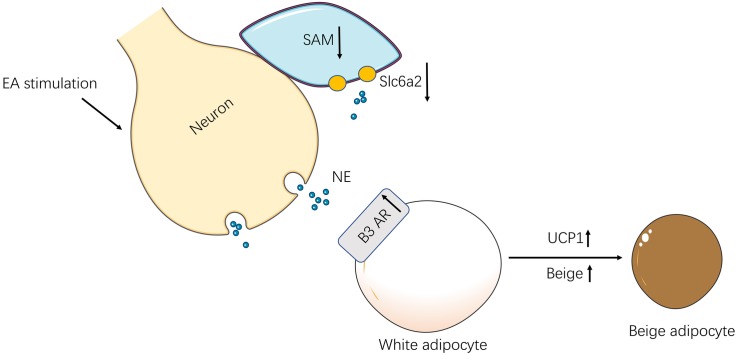
The schematic diagram of the neuro-immune cross-talk induced by EA. In obesity rats, EA stimulation activated the SNS in iWAT. For one thing, the SAMs decreased and reduced norepinephrine transporter-Slc6a2 by the excited sympathetic nerves. For another, the activation of sympathetic nerves increased β3 AR in iWAT and up-regulation UCP-1 which increased browning.

In conclusion, EA exerts its anti-obesity in rats by the SNS-immune cross-talk which promotes SNS activity and regulates immunologic balance (Figure 7).

## Data Availability Statement

All datasets generated for this study are included in the article/Supplementary Material.

## Ethics Statement

The animal study was reviewed and approved by Experimental Animal Ethics Committee of Nanjing University of Chinese Medicine.

## Author Contributions

BX, ZY, and MY conceived and designed the experiments. ML performed the experiments and wrote the manuscript. YH, MG, QL, QT, XW, and YW performed the experiments. ML, YH, and ZY analyzed the data. All authors read and approved the final version of article to be published.

## Conflict of Interest

The authors declare that the research was conducted in the absence of any commercial or financial relationships that could be construed as a potential conflict of interest.
